# Regenerating islet-derived protein 1 inhibits the activation of islet stellate cells isolated from diabetic mice

**DOI:** 10.18632/oncotarget.6163

**Published:** 2015-10-19

**Authors:** Wei Xu, Wei Li, Ying Wang, Min Zha, Honghong Yao, Peter M. Jones, Zilin Sun

**Affiliations:** ^1^ Department of Endocrinology, Zhongda Hospital, Institute of Diabetes, Medical School, Southeast University, Nanjing, China; ^2^ Department of Pharmacology, Medical School of Southeast University, Nanjing, China; ^3^ Diabetes Research Group, Division of Diabetes & Nutritional Sciences, Faculty of Life Sciences and Medicine, King's College London, London, UK

**Keywords:** regenerating islet-derived protein 1, exostosin-like glycosyltransferase 3, islet stellate cells, islet fibrosis, Pathology Section

## Abstract

Emerging evidence indicates that the islet fibrosis is attributable to activation of islet stellate cells (ISCs). In the present study, we compared the differences in biological activity of ISCs isolated from diabetic db/db and non-diabetic db/m mice, and the effects of the regenerating islet-derived protein 1 (Reg1) on ISC function. We showed that ISCs isolated from db/db mice were activated more rapidly than those from db/m mice during culture. Both Reg1 and its putative receptor exostosin-like glycosyltransferase 3 (EXTL3) were highly expressed by diabetic ISCs. Treatment with Reg1 inhibited migration, viability, and synthesis and secretion of Type I Collagen(Col-I), Type III Collagen(Col-III) and Fibronectin(FN) by diabetic ISCs, and this was associated with deactivation of the PI3K/Akt, MAPK/Erk1/2 signaling pathway in an EXTL3-dependent manner. In conclusion, our observations (i) confirmed the presence of fibrogenic stellate cells within pancreatic islets, which are prone to be activated in Type 2 diabetes, and (ii) revealed a potential role for Reg1 in preventing ISC activation.

## INTRODUCTION

Type 2 diabetes mellitus (T2DM) is a common group of metabolic disorders characterized by hyperglycemia, insulin resistance (IR) and β-cell dysfunction. Islet fibrosis promotes the progression of β-cell failure because it accelerates β-cell apoptosis [1] and reduces the capacity of β-cell proliferation, resulting in a reduced functional β-cell mass [2]. Previous studies have shown that the deposition of islet amyloid, activation of the renin-angiotensin system (RAS) and low-grade chronic inflammation all accelerate the development of islet fibrosis [3-5]. The pathogenesis of islet fibrosis has not yet been fully clarified, although it has been suggested that the activation of pancreatic stellate cells (PSCs) is a crucial mechanism initiating islet fibrosis in Type 2 diabetes [6]. Our recently research showed that PSCs may contribute to the efficient regulation of pancreatic development [7], which can be activated to proliferate and generate fibrotic extracellular matrix (ECM) by a range of environmental stimuli which are also associated with T2DM [8]. The process of stellate cell activation involves: (i) Proliferation and enlargement. (ii) Transformation into a myofibroblast-like phenotype and expression of the cytoskeletal marker protein α smooth muscle actin (α-SMA). (iii) Decreased expression of retinoid- containing fat droplets. (iv) Increased responsiveness to cytokines (such as transforming growth factor β (TGF-β) and platelet derived growth factor (PDGF)). (v) Increased synthesis and secretion of ECM components such as collagens and FN. Our recent *in vitro* study showed that a distinct population of islet stellate cells (ISCs) could be expanded from isolated islets. We observed the dynamic growth of ISCs by Live Cell Station and confirmed that they grew from the inside of the islet, rather than spreading from the edge of the islet. ISCs had a similar phenotype to PSCs, expressing α-SMA, Vimentin, glial fibrillary acidic protein (GFAP), and the ECM components, Col-I, Col-III and FN. However, Our study also provided evidence that ISCs are not identical to classical PSCs in terms of activation, proliferation, and motility [9]. Therefore, understanding the roles that participate in the process of islet fibrosis is critical for both understanding the mechanism and improving treatment of diabetes.

The pancreatic Regenerating Protein Product (Reg1) was first identified in pancreatic acinar cells, and Reg1 expression has been implicated in pancreatic cancer [10, 11], inflammatory bowel disease [12] and autoimmune diabetes [13]. Under normal conditions Reg1 is reported to be not expressed in rat [14, 15] or mouse [16]islets, but its expression is upregulated in mouse islets after induction of experimental diabetes with streptozotocin [17], and in human islets from patients with T2DM, where Reg1 expression levels correlate to the duration of diabetes [18]. Forced over-expression of Reg1 or the administration of exogenous Reg1 induces islet cell proliferation and leads to the amelioration of diabetes [19].

Reg1 inhibits PSC proliferation and migration, and reduces the synthesis and secretion of Col-I, FN, matrix metallopeptidase (MMP)-1 and MMP-2, and the tissue inhibitors (TI) of metalloproteinases TIMP-1 and TIMP-2 [20]. The putative receptor through which Reg1 exerts its biological effects has been identified as having 97% homology with the human multiple EXTL3 [21, 22], which is highly expressed in mouse islets during embryonic development with reduced expression in adult islets [23], consistent with a role for Reg1/EXLT3 in β-cell expansion and regeneration.

The individual (patho)physiological roles of Reg1/EXTL3 and PSCs have been investigated extensively but little is known about interactions between Reg1 and ISCs in the regulation of islet fibrotic responses. In this study we have therefore characterized the phenotype of ISCs isolated from normal and diabetic mouse islets, and investigated the effects of Reg1 on ISC function.

## RESULTS

### Expression of Reg1 and EXTL3 in pancreas, islets and ISCs

**Figure 1:**
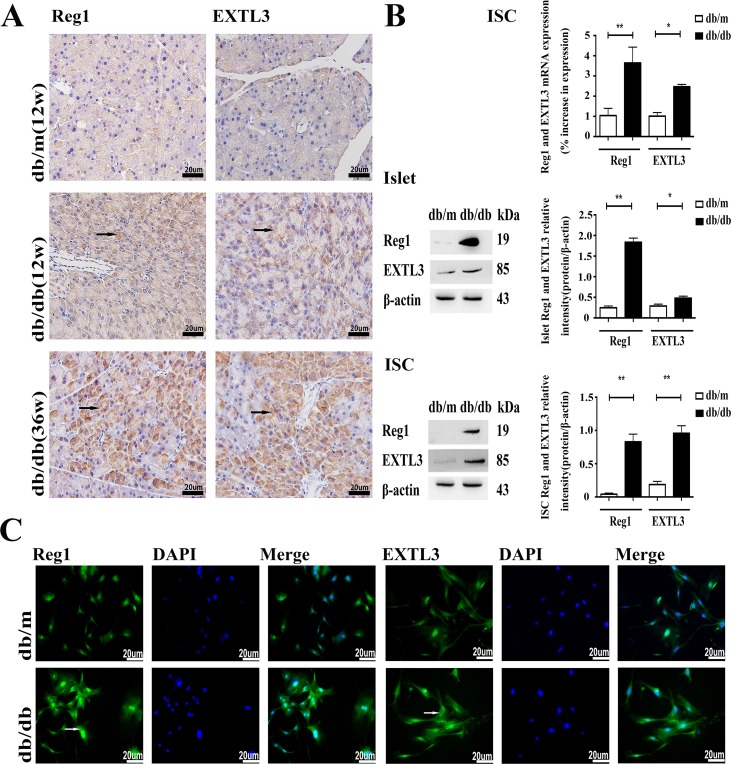
Expression of Reg1 and EXTL3 in pancreas, islets and ISCs **A.** Wax-embedded sections of db/db and db/m mouse pancreases showing expression of Reg1 and EXTL3 by immunohistochemistry. Scale bar = 20μm. **B.** Quantification of Reg1 and EXTL3 mRNA and protein by qRT-PCR and Western blotting respectively in islets and ISCs. Data are expressed as mean ± SE (n = 3), * *P* < 0.05, ** *P*< 0.01, db/db ISCs compared with db/m ISCs. **C.** Immunofluorescent staining of ISCs by Reg1 and EXTL3 antibodies (green). Scale bar = 20μm.

Reg1 and EXTL3 protein expression were detectable by immunohistochemistry in pancreatic sections, as shown in Figure 1A. Pancreas from normoglycemic db/m mice showed a few lightly immunostained Reg1^+^ and EXTL3^+^ cells. In contrast, pancreas from hyperglycemic db/db mice contained numerous large, heavily-immunostained Reg1^+^ and EXTL3^+^ cells throughout the pancreatic tissue. Expression of Reg1 and EXTL3 mRNAs and proteins was confirmed by qRT-PCR and Western blotting analysis using extracts of isolated islets and ISCs from db/db mice and db/m mice, as shown in Figure 1B. Both mRNA and protein levels of Reg1 and EXTL3 were much lower in control db/m islets and ISCs than in the equivalent tissues isolated from diabetic db/db mice, in which expression levels were much higher (Figure 1B). These observations were confirmed by the immunofluorescence microscopy measurements shown in Figure 1C, in which both Reg1 and EXTL3 immunoreactivities were higher in ISCs isolated from db/db mice than in ISCs from db/m mice. Together these data demonstrate that Reg1 and EXTL3 are much more highly expressed in pancreatic tissues from diabetic mice than from control mice.

### Differential activation, migration, viability and ECM expression of ISCs isolated from control and diabetic mice

**Figure 2:**
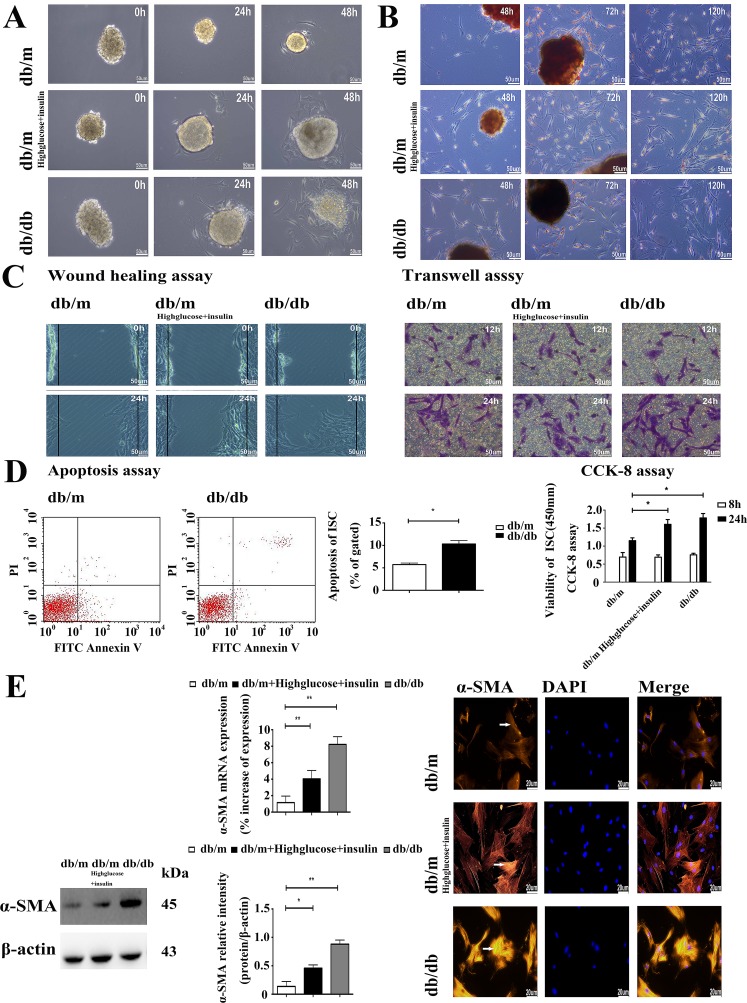
The diabetic environment promotes the activation of ISCs **A.** Light microscopy micrographs demonstrating faster rates of ISC outgrowth from diabetic islets. Scale bar = 50μm. **B.** Oil red “O” staining of lipid droplets in cytoplasm from ISCs shows faster activation and loss of lipid droplets in diabetic ISCs. Scale bar = 50μm. **C.** Wound healing assay of migration rate shows greater migration of diabetic ISCs. Transwell assay of migration rate shows more diabetic ISCs migrating across the filter. Scale bar = 50μm. **D.** CCK-8 assay of viability in ISCs. Data are expressed as mean ± SE (n = 15), * *P*< 0.05, ** *P*< 0.01. Apoptosis assay of apoptosis in ISCs. Data are expressed as mean ± SE (n = 3), * *P*< 0.05, ** *P*< 0.01. **E.** qRT-PCR and Western blotting of α-SMA mRNA and protein expression in ISCs. Data are expressed as mean ± SE (n = 3), * *P* < 0.05, ** *P*< 0.01. Immunofluorescent staining of ISCs by α-SMA antibody. Scale bar = 20μm.

Diabetic ISCs had a different function phenotype to those isolated from control db/m islets. Thus, the rate of ISCs outgrowth was markedly faster from db/db islets and db/m islets cultured in high glucose and insulin medium when compared to those from db/m mice, as shown in Figure 2A, and the rate of activation from the quiescent state, as judged by the loss of Red-O stained fat droplets (Figure 2B and Supplemental Material S1) was more rapid in ISCs isolated from the db/db islets and db/m islets cultured in high glucose and insulin medium. We used two different *in vitro* assays - wound healing and transwell migration - to compare the migration rates of db/m ISCs, db/m ISCs cultured in high glucose and insulin medium and db/db ISCs. Both assays showed that db/db ISCs and db/m ISCs cultured in high glucose and insulin medium had a significantly greater migration rate than control db/m ISCs (Figure 2C and Supplemental Material S2). Similarly, db/db ISCs had significantly higher rates of apoptosis and viability than control db/m ISCs (Figure 2D), consistent with the enhanced rates of migration and activation of the ISCs isolated from the diabetic db/db islets.

**Figure 3:**
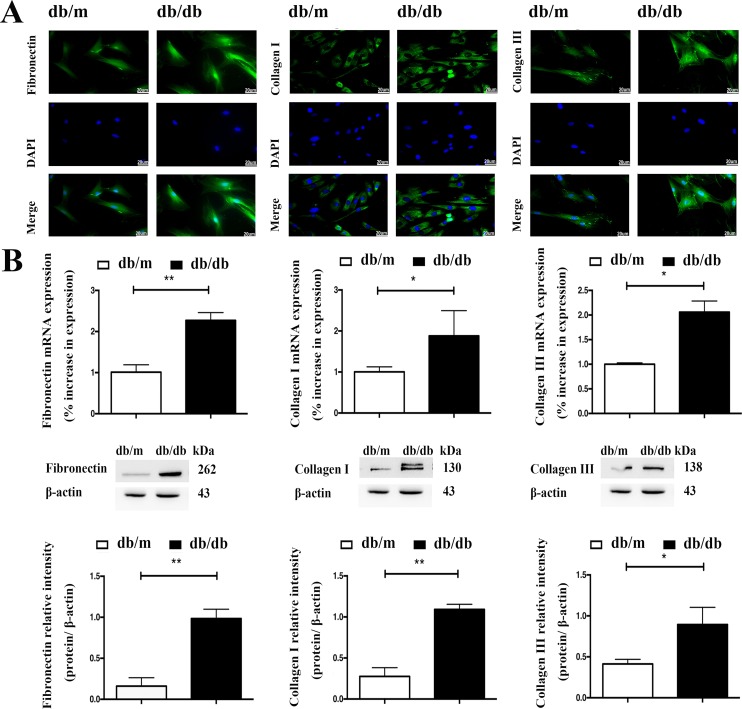
The diabetic environment enhances ECM expression by ISCs **A.** Immunofluorescent staining of ISCs for Col-I, Col-III and FN. Scale bar = 20μm. **B.** qRT-PCR and Western blotting measurements of mRNA and protein expression of Col-I, Col-III and FN in ISCs. Data are expressed as mean ± SE (n = 3), * *P*< 0.05, ** *P*< 0.01, db/db ISCs vs. db/m ISCs.

In accordance with their activation status, ISCs isolated from the diabetic mouse islets and db/m ISCs cultured in high glucose and insulin medium also expressed more α-SMA than control ISCs, as assessed by qRT-PCR measurements of elevated levels of α-SMA mRNA and Western blotting (Figure 2E) and immunohistochemical measurements of α-SMA protein (Figure 2E). Similarly, qRT-PCR, Western blotting and immunofluorescence microscopy demonstrated that db/db ISCs expressed higher levels of Col-I, Col-III and FN than control ISCs, as shown in Figure 3A and 3B. Together, these data demonstrate that ISCs isolated from a diabetic environment exhibit the characteristics of activated stellate cells in terms of migration, viability, ECM deposition and activation than ISCs from a normoglycemic environment, consistent with ISC activation contributing to the islet pathology associated with T2DM.

### Reg1 inhibits the activation of ISCs in db/db mouse via its receptor EXTL3

**Figure 4:**
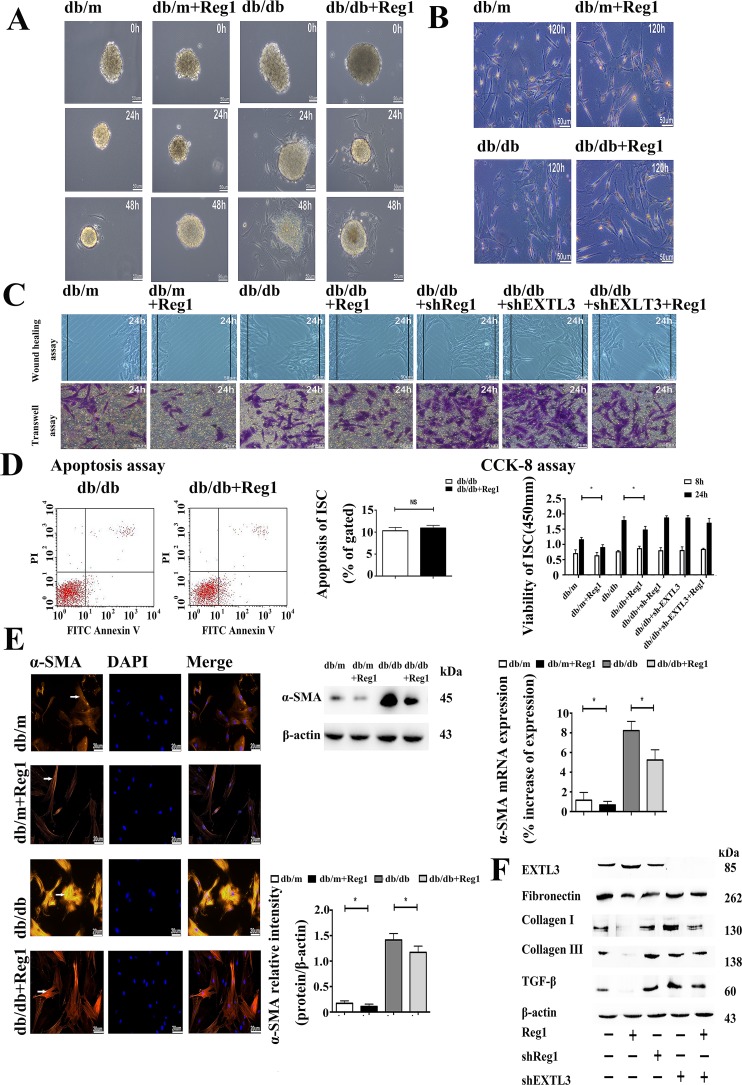
Reg1 inhibits the activation of ISCs via its receptor EXTL3 **A.** Light microscopy micrographs demonstrating rates of ISC outgrowth from islets treated with Reg1. Scale bar = 50μm. **B.** Oil red “O” staining of lipid droplets in cytoplasm from ISCs shows slower activation and loss of lipid droplets after treated with Reg1. Scale bar = 50μm. **C.** Both the wound healing assay and the transwell migration assay showed reductions in migration of ISCs treated with rhReg1 (100ng/ml), which was reversed by down-regulation of EXTL3. Scale bar = 50μm. **D.** CCK-8 assay of viability in ISCs treated with rhReg1, sh-Reg1, sh-EXTL3 and sh-EXTL3+rhReg1. Data are expressed as mean ± SE (n = 15), * *P* < 0.05, ** *P*< 0.01. Apoptosis assay of apoptosis in ISCs. Data are expressed as mean ± SE (n = 3), * *P* < 0.05, ** P< 0.01. **E.** qRT-PCR and Western blotting of α-SMA mRNA and protein expression in ISCs treated with Reg1. Data are expressed as mean ± SE (n = 3), * *P* < 0.05, ** *P*< 0.01. Immunofluorescent staining of ISCs by α-SMA antibody. Scale bar = 20μm. **F.** Western blotting of db/db ISCs treated with rhReg1, sh-Reg1, sh-EXTL3 and sh-EXTL3+rhReg1 by EXTL3, Col-I, Col-III, FN and TGF-β antibody. Data were expressed as mean ± SE (n = 3), * *P* < 0.05, ** *P* < 0.01.

We used shRNA-mediated knockdown of Reg1 and EXTL3 expression in db/db ISCs. Exposured to shRNAs directed against Reg1 and EXTL3 caused a significant reduction in the expression of Reg1 and EXTL3 mRNAs by 72 hours when compard to ISCs transfected with control non-targeting shRNAs (Supplemental Material S3). The knockdown of Reg1 and EXTL3 mRNA was maintained and immunoblot analysis confirmed that Reg1 and EXTL3 protein was also greatly reduced at 72 hours post-transfection (Supplemental Material S3). We treated islets with rhReg1 to investigate the effects of Reg1 on the rate of ISCs outgrowth. The presence of rhReg1 reduced the rate of ISCs outgrowth from db/m and db/db islets were markedly slower when compared to control, as shown in Figure 4A.

To investigate the effects of Reg1 on ISC activation we treated ISCs with rhReg1, which inhibited their activation as assessed by a significantly decreased loss of Red-O stained fat droplets (Figure 4B and Supplemental Material S4), migration (Figure 4C and Supplemental Material S5), viability (Figure 4D), expression of α-SMA (Figure 4E) and reduced synthesis and secretion of Col-I, Col-III and FN (Figure 4F and Supplemental Material S6) with no change in the levels of apoptosis (Figure 4D). Conversely, down-regulation of Reg1 and EXTL3 expression in db/db ISCs by using shRNA increased the activation of db/db ISCs, assessed using the same parameters (Figure 4C, 4D, 4F). In accordance with EXTL3 being the ISC receptor for Reg1, shRNA-induced down-regulation of EXTL3 abolished the effects of exogenous Reg1 to reduce activation of db/db ISCs, as shown in Figure 4C, 4D, 4F. Together, these results demonstrate that Reg1 inhibits the activation of ISCs via its receptor EXTL3.

### Intracellular mechanism of action of Reg1 in the inhibition of ISC activation

**Figure 5:**
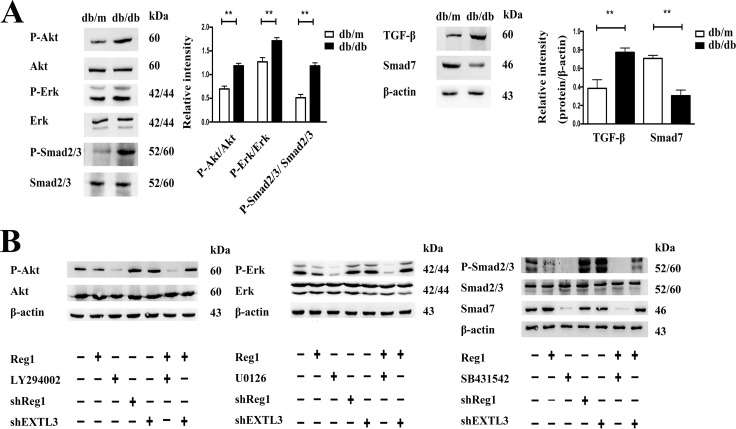
Intracellular mechanism of action of Reg1 in the inhibition of ISC activation **A.** Western blotting of ISCs by Erk1/2, P-Erk1/2, Akt, P-Akt, Smad2/3, P-Smad2/3, Smad7, TGF-β antibody. Data are expressed as mean ± SE (n = 3), * *P* < 0.05, ** *P* < 0.01. **B.** Western blotting of db/db ISCs treated with rhReg1, U0126 (10uM), LY-294002 (10uM), SB431542 (10uM), sh-Reg1, sh-EXLT3 and sh-EXTL3+Reg1 by Erk1/2, P-Erk1/2, Akt, P-Akt, Smad2/3, P-Smad2/3 and Smad7 antibody.

To identify potential intracellular signaling cascades involved in the Reg1/EXTL3 modulation of ISCs activation we used a combination of immunoblot and pharmacological inhibitors to identify specific signaling elements. Under basal conditions, db/db ISCs had higher levels than control ISCs of phosphorylation, and thus activation, of Erk1/2, Akt, Smad2/3 and reduced expression of Smad7, as shown in Figure 5A. Incubation of db/db ISCs with rhReg1, U0126 (Erk pathway inhibitor), LY-294002 (PI3K-Akt pathway inhibitor), SB431542 (Smad pathway inhibitor) caused a significant decrease in phosphorylation of Erk1/2, Akt and Smad2/3 (Figure 5B and Supplemental Material S7). In contrast, treatment with rhReg1 or the pharmacological inhibitors had no effects on total Erk1/2, Akt and Smad2/3 expression, confirming that the effects observed were on the activation of the intracellular signaling pathways. Sh-RNA induced down-regulation of Reg1 or EXTL3 caused small increases in basal phosphorylation of Erk1/2, Akt, and Smad2/3 (Figure 5B and Supplemental Material S7) but EXTL3 downregulation blocked the effects of exogenous rhReg1 to inhibit phosphorylation of Erk1/2 and Akt, although the ability of rhReg1 to inhibit phosphorylation of Smad2/3 was not blocked by EXTL3 down-regulation (Figure 5B and Supplemental Material S7). Together, these observations suggest that Reg1 acts via EXTL3 to inhibit the Erk and Akt pathways in db/db ISCs, but through another unidentified mechanism to influence TGF-β/ Smad pathway activity.

## DISCUSSION

Islet fibrosis contributes to the progression of beta cell failure by accelerating beta cell destruction in a diabetic environment. Emerging evidence indicates that the islet fibrosis is attributable to activation of islet stellate cells (ISCs).

We have previously reported that isolated islets contain a population of stellate cells, which are phenotypically similar but not identical to PSCs. In common with PSCs these ISCs expressed Vimentin, α-SMA and GFAP, and synthesised and secreted the ECM components. ISCs differed from PSCs by having significantly reduced rates of proliferation and migration *in vitro*. Given the anatomical location of ISCs, and the importance of fibrosis in β-cell dysfunction we suggested that ISCs maybe contribute significantly to islet fibrosis in T2DM. In the current study we have extended these observations to show that the functional phenotype of ISCs is modified by the diabetic environment *in vitro* and *in vivo*. This activation of the diabetic ISCs was associated with the molecular markers previously identified for PSC activation [26], most importantly increased viability and migration and the enhanced synthesis and deposition of ECM components, all of which associated with the fibrotic response. These observations are consistent with ISCs responding to the diabetic environment with increased activation potential, leading to islet fibrosis and inhibition of normal β-cell function and/or survival. Understanding the mechanisms leading to the activation of ISCs in the diabetic environment may therefore identify potential therapeutic targets to prevent the progression to fibrosis in T2DM.

Our studies have identified the Reg1/EXTL3 system as one mechanism through which the diabetic environment influences ISC function. Reg1 protein is a well-known component of pancreatic exocrine juice, and was first identified in patients with chronic pancreatitis, where it was initially named as human Pancreatic Stone Protein (PSP) [27]. PSP was later renamed Pancreatic Thread Protein (PTP) [28]and later as pancreatic Reg1 when it was shown to be expressed in regenerating islet cells following a near total pancreatectomy [29, 30]. Reg1 expression is induced during ductal proliferation, β-cell growth and islet regeneration [31, 32], and exogenous Reg1 protein has mitogenic effects on ductal cells and β-cells [33, 34], reverses diabetes in some animal models of β-cell failure [35, 36]. Reg1 expression has also been implicated in the islet inflammation and fibrosis associated with the development of spontaneous diabetes in GK rats [37]and we have previously reported that Reg1 expression is significantly up-regulated in patients with T2DM, and that Reg1 levels are related to the duration of the TDM [18].

In the present study we have shown that Reg1 and its receptor EXTL3 are expressed in ISCs, albeit at a relatively low level under normal conditions, which may explain why these Reg1/EXTL3 positive cells within islets have not been detected in previous studies [14-16]. However, the expression of both molecules is highly upregulated in ISCs isolated from diabetic db/db mice and in db/m ISCs cultured in high glucose and insulin medium, demonstrating that the diabetic environment increased their expression in ISCs. In accordance with this, exogenous Reg1 inhibited the activation of db/m ISCs and the shRNA-mediated down-regulation of endogenous Reg1 in db/db ISCs increased their activation, demonstrating that the increased rate of activation of the db/db ISCs was caused by the diabetic environment regulating the expression of Reg1/EXTL3. Thus, exposing ISCs to a hyperglycemic, inflammatory pre-diabetic environment leads to upregulation of Reg1/EXTL3 to maintain them in a quiescent state. With disease progression, ISCs produce more Reg1 to protect against the hostile environment but the protective effects of Reg1 becomes overwhelmed, causing the ISCs to become activated to a “fibrotic” phenotype and to lay down excessive ECM resulting in islet fibrosis. Our observations suggest that the Reg1/EXTL3 system exerts a suppressive effect on ISC activation, and thus on potential fibrotic responses to environmental factors. This conclusion is supported by our demonstration that exogenous Reg1 enhanced EXTL3 expression and reduced the activation of db/db ISCs. The effects of Reg1 were mediated through EXTL3 because they were abolished when we down-regulated EXTL3 expression in the db/db ISCs using shRNA. Thus, the results of our current study suggest that Reg1 acts via EXTL3 suppress activation of ISCs, and subsequent islet fibrosis.

Our study has also provided some insight into the intracellular mechanisms through which the Reg1/EXTL3 system inhibits ISC function. We have demonstrated for the first time that Reg1 reduced the phosphorylation and thus activation of Akt, Erk1/2 and Smad2/3, which is elevated in ISCs as a consequence of the diabetic environment. The identification of the Erk, Akt and Smad signaling pathways as targets for Reg1 in ISCs is in accordance with previous reports in other cell types. For example, the PI3K/Akt pathway is associated with cell migration, but not proliferation in PSCs [38, 39], consistent with our demonstration that the Akt pathway is activated in diabetic ISCs, and that exogenous Reg1 can reduce Akt activation and diabetic ISC migration. The Erk/MAPK cascade is involved in a variety of cellular processes, including cell proliferation, cell survival, apoptosis, and cytokine production [40], and previous studies have shown that Erk activation mediates proliferation and α-SMA expression in PSCs [41], consistent with our observations that Reg1 inhibits phosphorylation and activation of Erk1/2 in db/db ISCs and that this is associated with their reduced viability. It has been reported that rat PSCs exhibited increased TGF-β/Smad pathway activity, resulting in increased ECM synthesis, increased activation and α-SMA expression [42], and Smad2, Smad3, and Smad4 have been implicated in PSCs function [43], consistent with our demonstration that the TGF-β/Smad pathway is activated in diabetic ISCs, Reg1 decrease the expression of ECM through inhibition of the TGF-β/Smad pathway, returning levels of phospho-Smad2/3 to those of normoglycemic ISCs. Although our data demonstrate that EXTL3 is a receptor for Reg1 in ISC signaling via the Reg1-EXTL3-Akt and Reg1-EXTL3-Erk pathways, the effect of EXTL3 down-regulation on Reg1 signalling via phospho-Smad2/3 was incomplete, suggesting that Reg1 influences TGF-β/Smad signaling in ISCs via an additional, and as yet unidentified, pathway independent of EXTL3. Although these *in vitro* findings support our hypothesis that Reg1 is important for suppression of ISCs activation in a diabetic environment further *in vivo* studies are required, perhaps using transgenic models, to confirm the importance of this process in the pathogenesis of Type 2 diabetes.

In summary, we have identified a distinct population of stellate cells in islets with a phenotype distinct from standard PSCs, and demonstrated that this population of ISCs is activated towards a fibrotic phenotype by chronic exposure to a diabetic environment, suggesting that these cells participate in the development of islet fibrosis, and thus are central to the pathogenesis of T2DM. ISC activation is inhibited by Reg1, which acts primarily through EXTL3 to prevent diabetes-induced activation of the ISC fibrotic phenotype. These studies demonstrate that Reg1 can not only promote islet regeneration, but can also decrease the activation of ISCs and delay the development of islet fibrosis, which may provide new targets for clinical drug development to prevent or treat T2DM.

## MATERIALS AND METHODS

### Animals

The study was reviewed and approved by the Animal Care and Use Committee of Southeast University. Specific-pathogen-free male C57BL/KsJ-db/db mice (aged 8-12 weeks) and age- and sex-matched male lean littermate C57BL/KsJ-db/m mice (aged 8-12 weeks) were used as controls. All animals were purchased from the Model Animal Research Center of Nanjing University (Nanjing, China).

### Isolation and culture of mouse islet stellate cells

Mouse islets were isolated from db/m and db/db mice (12 weeks old and 6 mice each group, the blood glucose levels were greater than 13mmol/l) by type IV collagenase (1 mg/ml; Sigma, CA, USA) digestion of the exocrine pancreas followed by purification on Histopaque (Sigma, CA, USA) density gradients. The isolated islets were maintained in culture for up to 48 hours at 37°C (95% air/5% CO_2_) [24], with the majority of islets attaching to the dish within 3 to 7 days. Cultures were re-fed when the majority of islets had attached and thereafter as needed to replenish nutrients and remove debris. “Passage 0” is defined as 10 to 14 days after the islets were placed in culture at a time when the cultures were nearly confluent with stellate cells. Beginning at passage 0, cells were harvested with trypsin and sub-cultured (1:2) every 3-4 days. Cells were maintained in Dulbecco's modified Eagle's medium (DMEM)/Ham's F12 (1: 1 v/v) (Sigma, CA, USA) containing 10% (v/v) fetal calf serum (FCS) and used from passage 3-8.

### Immunohistochemistry

Pancreases (12, 36-week old) were perfused and fixed in 4% paraformaldehyde in 0.1 M PBS for 24 hours at 4°C, paraffin embedded and sectioned (4 μm). Paraffin-fixed tissue sections were blocked with 5% bovine serum albumin (BSA) for 30 min, incubated overnight at 4°C with rabbit anti-mouse Reg1 (1:200, Abcam, Cambridge, UK) or rabbit anti-mouse EXTL3 (1:200, Santacruz, Dallas, USA), then washed and incubated with HRP-linked anti-rabbit serum at room temperature for 30 min. The sections were then developed with DAB, counterstained with hematoxylin, and examined using an Olympus BX40 microscope.

### Immunofluorescence Microscopy of Reg1, EXTL3, α-SMA, FN, Col-I and Col-III

Immunofluorescence microscopy was performed as described previously [9] to evaluate differences between ISCs isolated from db/m and db/db mice in the expression of Reg1, EXTL3, α-SMA, FN, Col-I and Col-III (1:200, Abcam, Cambridge, UK). All immunocytochemical analyses were performed in triplicate.

### Oil red O staining

Islets and ISCs were either cultured alone, or in medium supplemented with D-glucose (25mmol/l) and insulin (100nM) (high glucose and insulin medium), or with rhReg1 (100ng/ml) for 24 hours unless otherwise specified. Oil Red O Staining (Sigma, CA, USA) was performed as described previously [9].

### Determination of cell viability

Cell viability was assessed directly by WST-8 assay (Sigma, CA, USA) as described previously [9]. ISCs were either cultured for alone, or with D-glucose and insulin (high glucose and insulin medium), or with shRNA, or with rhReg1 for 24 hours unless otherwise specified. All experiments were performed in quintuplicate on three separate occasions (*n* = 15). Three independent experiments were performed in triplicate.

### Detection of cell migration

### Wound healing

ISCs were seeded in 6-well culture plates and grown for 24 hours to reach confluence. ISCs were cultured as described above. After starvation with serum-free medium overnight, Then wound healing assay was performed as described previously [9].

### Transwell assay

ISCs were seeded in transwells (BD 353097, 8 mm pores), ISCs were cultured as described above and the transwell assay was performed as described previously [9].

### Apoptosis assay

An annexin V-fluorescein isothiocyanate (FITC)/propidium iodide (PI) apoptosis detection kit was used to analyze cell apoptosis as described previously [9].

### Quantitative real-time polymerase chain reaction (qRT- PCR)

ISCs were seeded into Nunclon™ 35mm petri dishes, and either cultured alone, or with high glucose and insulin medium, or with shRNA, or with rhReg1 for 24 hours unless otherwise specified. After 24 hours, total RNA was extracted from ISCs using TRIzol (Invitrogen, NY, USA), and 1 μg of total RNA was reverse transcribed into first-strand cDNA by using a reverse transcription reagent kit (TaKaRa BIO, Otsu, Japan) according to the manufacturer's protocol. qRT-PCR was performed using the SYBR® Green real-time PCR kit (TaKaRa BIO, Otsu, Japan). qRT-PCR was performed on the ABI StepOnePlus Real-Time PCR system (Applied Bio-systems, Foster City, CA, USA). All quantifications were performed with U6 as the internal standard. The PCR primer sequences were as follows:

Reg1, 5′-TAACAGTTCCAATCGTGGCTAC -3′ (sense) and 5′-GGGCATCACAGTTCTCATCCT -3′ (antisense);

EXTL3, 5′-TGCCCTGGAATGAGATAGAGAC -3′ (sense) and 5′-TGATGTGGGAGACAAGGAAGTT -3′ (antisense);

α-SMA, 5′-CAGCAAACAGGAATACGACGAA -3′ (sense) and 5′-AACCACGAGTAACAAATCAAAGC -3′ (antisense);

FN, 5′- GCAAGAAGGACAACCGAGGAAA -3′ (sense) and 5′-GGACAGCAGTGAAGGAGCCAGA -3′ (antisense);

Col-I, 5′-GTCAGACCTGTGTGTTCCCTACTCA -3′ (sense) and 5′-TCTCTCCAAACCAGACGTGCTTC -3′ (antisense);

Col-III, 5′-GGACCAGGCAATGATGGAAAAC -3′ (sense) and 5′-GGACCAGGGAAACCCATGACA -3′ (antisense);

β-actin, 5′-GAGAGGGAAATCGTGCGTGACA -3′ (sense) and 5′-ACCCAAGAAGGAAGGCTGGAAA -3′(antisense).

Relative gene expression was analyzed using the 2-ΔΔCT method, and the results were expressed as extent of change with respect to control values. qRT-PCR experiments were replicated at least 3 times.

### Western blotting analysis

ISCs were seeded into Nunclon™ 35mm petri dishes and either cultured alone, or in the presence of shRNA, or rhReg1, or U0126 (10μM, Sigma, CA, USA), or LY294002 (10μM, Sigma, CA, USA), or SB431542 (10μM, Selleck, Houston, USA) for 24 hours unless otherwise specified. After 24 hours, ISCs were lysed with ice-cold lysis buffer supplemented with protease inhibitors (Roche, Basel, Switzerland). After protein content determination and separation with 12%(w/v) SDS-PAGE, Western blotting was performed, as described previously [25], using antibody against primary antibody in 2.5% non-fat dried milk in Tris-buffered saline with Tween-20 (TBST) buffer. The primary antibody were as follows: Reg1 (1:3000, Abcam, Cambridge, UK), EXTL3 (1:200, Santacruz, Dallas, USA), α-SMA (1:1000, Abcam, Cambridge, UK), Col-I (1:5000, Abcam, Cambridge, UK), Col-III (1:5000, Abcam, Cambridge, UK), FN (1:5000, Abcam, Cambridge, UK), TGF-β, Akt, P-Akt, Erk, P-Erk, Smad2/3, P-Smad2/3, Smad7 (1:1000, Cell Signaling, MA, USA), β-Actin (1:2000, Sigma, CA, USA).

### shRNA preparation and targeting gene knockdown

Specific shRNAs and control shRNA were designed and synthesised by HANBIO (Shanghai, China). Blast search was performed with the National Center for Biotechnology Information (NCBI) database to ensure that the shRNA constructs were targeting only mouse Reg1 or EXTL3. The oligonucleotides were annealed and cloned into the pHBLV-U6-ZsGreen-Puro as the manufacturer described. 10μg of pHBLV-U6-ZsGreen-Puro constructs containing shRNAs, 10μg of packaging plasmid psPAX2 (Addgene), and 10μg of envelope plasmid pMD2.G (Addgene) were used to transfect HEK293T cells by the calcium phosphate precipitation method. After 48 hours lentiviruses containing targeted gene shRNA were collected and used to transfect ISCs according to the manufacturer's instructions.

### Statistical analysis

Quantitative data were expressed as mean±SEM. The statistical significance was determined by analysis of variance followed by Bonferroni's T test for multiple comparisons, and differences were considered significant when *P* < 0.05.

## SUPPLEMENTARY MATERIAL FIGURES


