# Supplementary material for: Regenerating islet-derived protein 1 inhibits the activation of islet stellate cells isolated from diabetic mice
Source: Oncotarget. 2015 Oct 19;6(35):37054–65. doi: 10.18632/oncotarget.6163 (PMC4741915; doi:10.18632/oncotarget.6163)
Supplement: Supplementary file 1 [file oncotarget-06-37054-s001.pdf]

Regenerating islet-derived protein 1 inhibits the activation of islet stellate cells isolated from diabetic mice

Supplemental Material

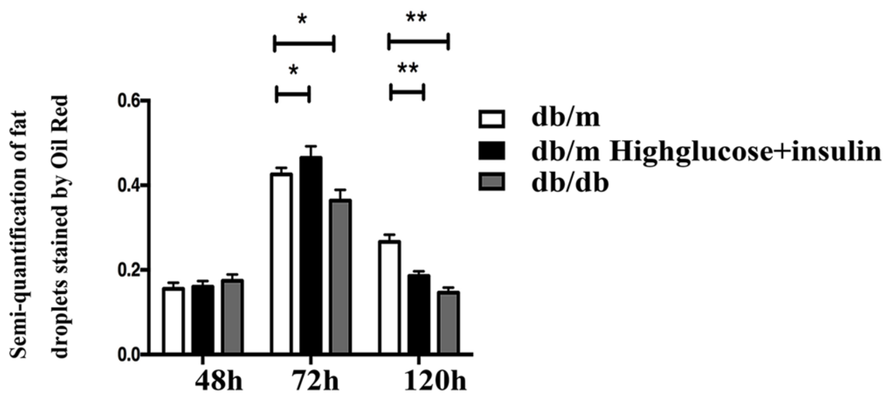

Supp Figure 1: Oil red "O" staining of lipid droplets in cytoplasm from ISCs shows faster activation and loss of lipid droplets in diabetic ISCs.

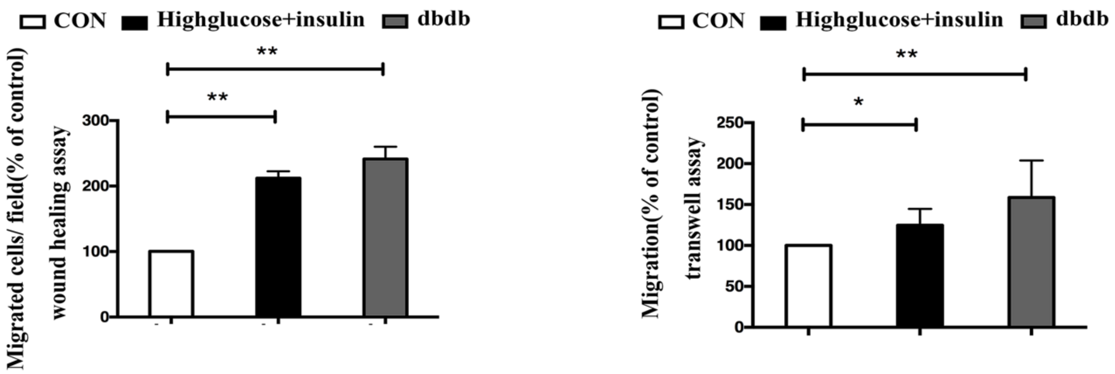

Supp Figure 2: Wound healing assay of migration rate shows greater migration of diabetic ISCs. Transwell assay of migration rate shows more diabetic ISCs migrating across the filter.

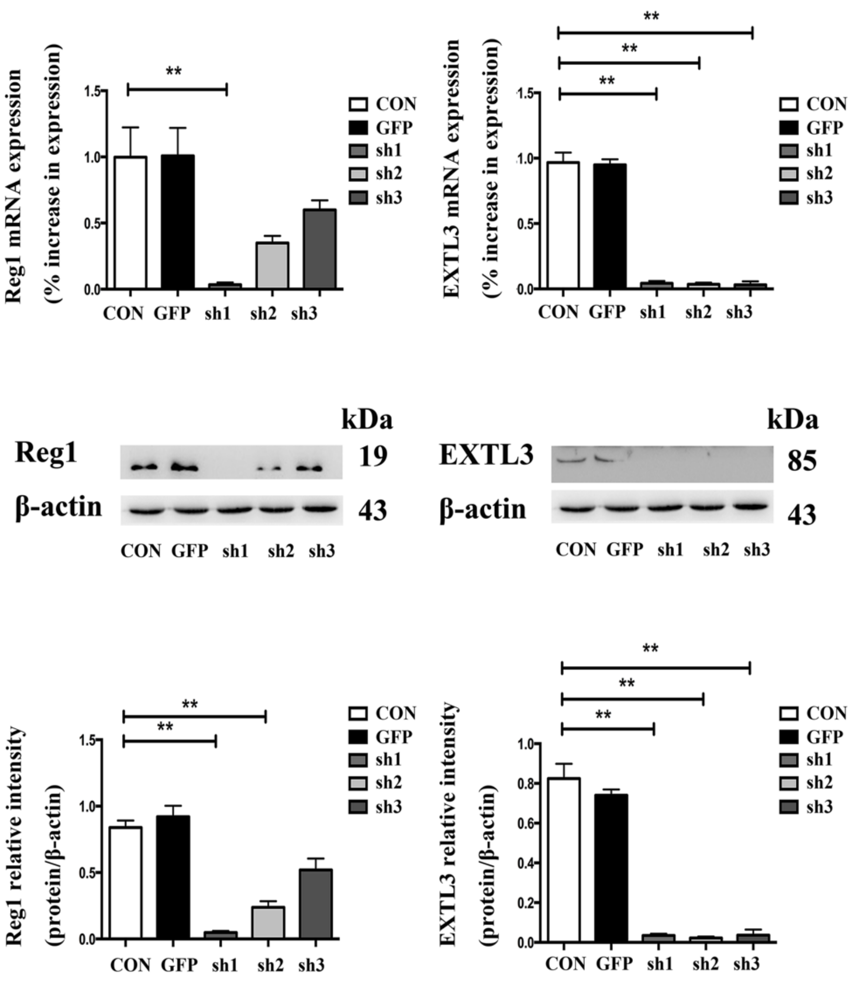

Supp Figure 3 : Western blotting of db/db ISCs treated with sh-Reg1, sh-EXTL3 by Reg1 and EXTL3 antibody. Data were expressed as mean ± SE (n = 3), * $P < 0.05$, ** $P < 0.01$.

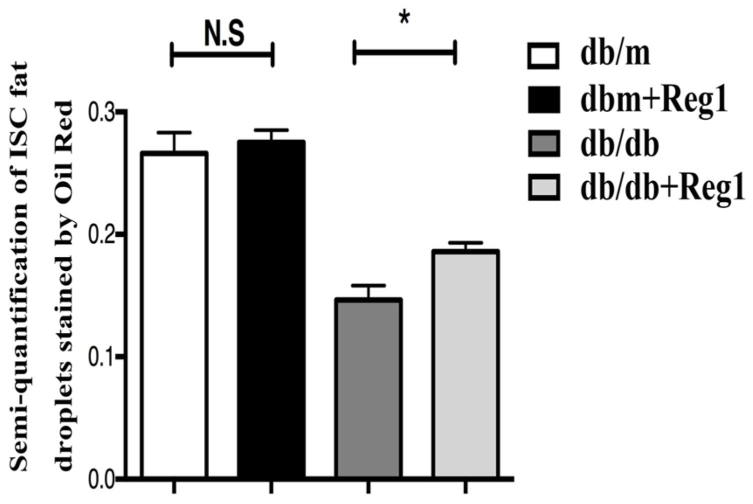

Supp Figure 4: Oil red “O” staining of lipid droplets in cytoplasm from ISCs shows slower activation and loss of lipid droplets after treated with Reg1.

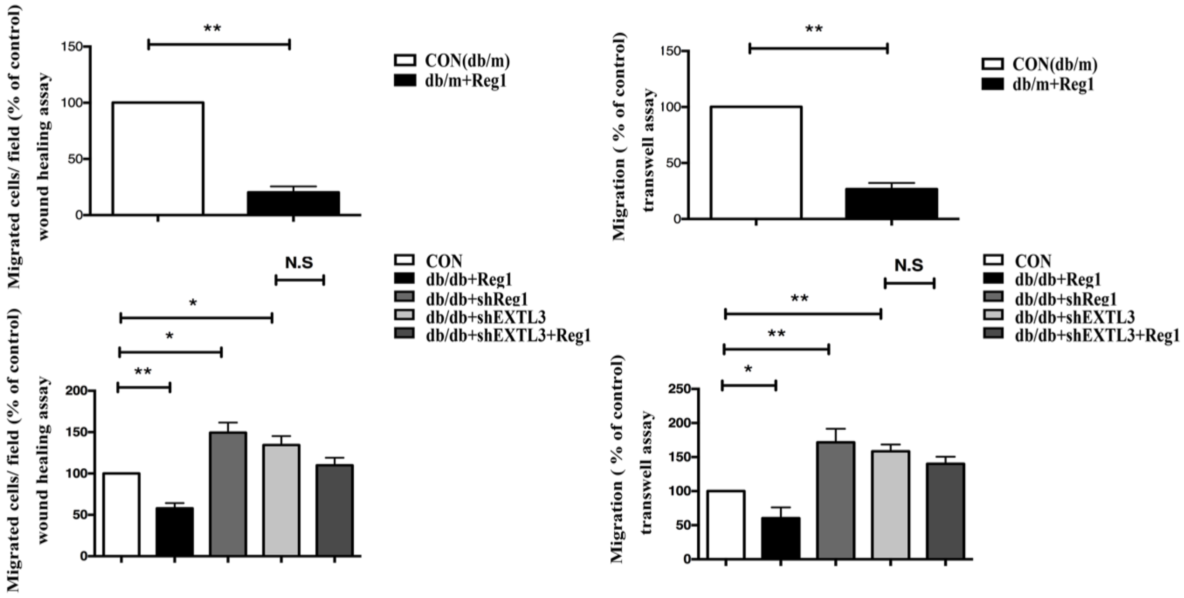

Supp Figure 5: Both the wound healing assay and the transwell migration assay showed reductions in migration of ISCs treated with rhReg1 (100ng/ml), which was reversed by down-regulation of EXTL3.

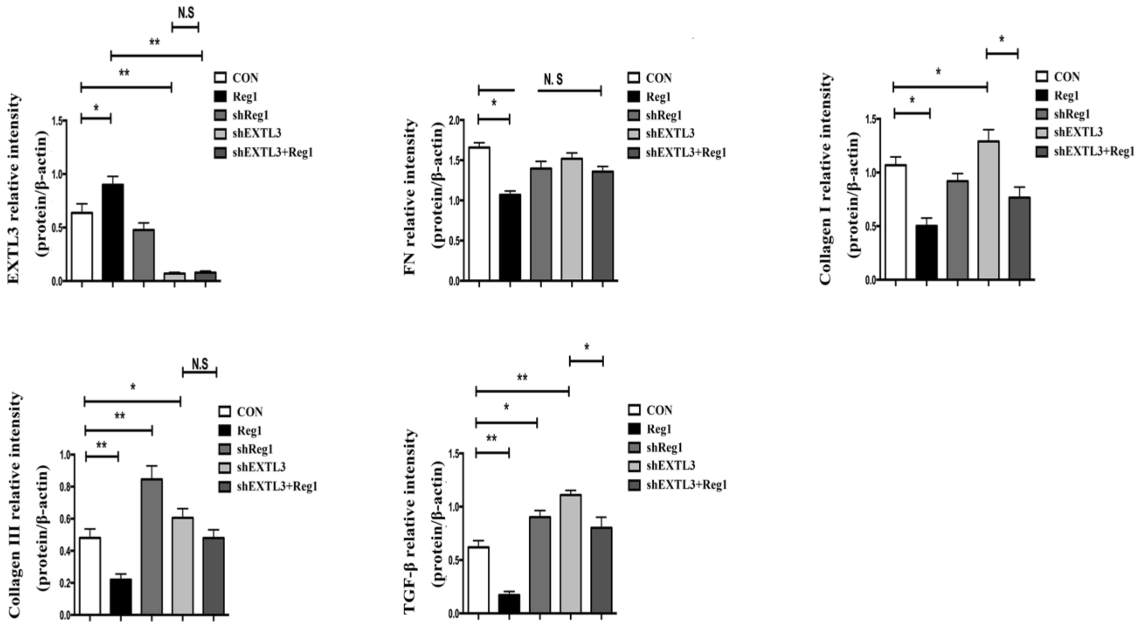

Supp Figure 6: Western blotting of db/db ISCs treated with rhReg1, sh-Reg1, sh-EXTL3 and sh-EXTL3+rhReg1 by EXTL3, Col-I, Col-III, FN and TGF-β antibody. Data were expressed as mean ± SE (n = 3), * P < 0.05, ** P < 0.01.

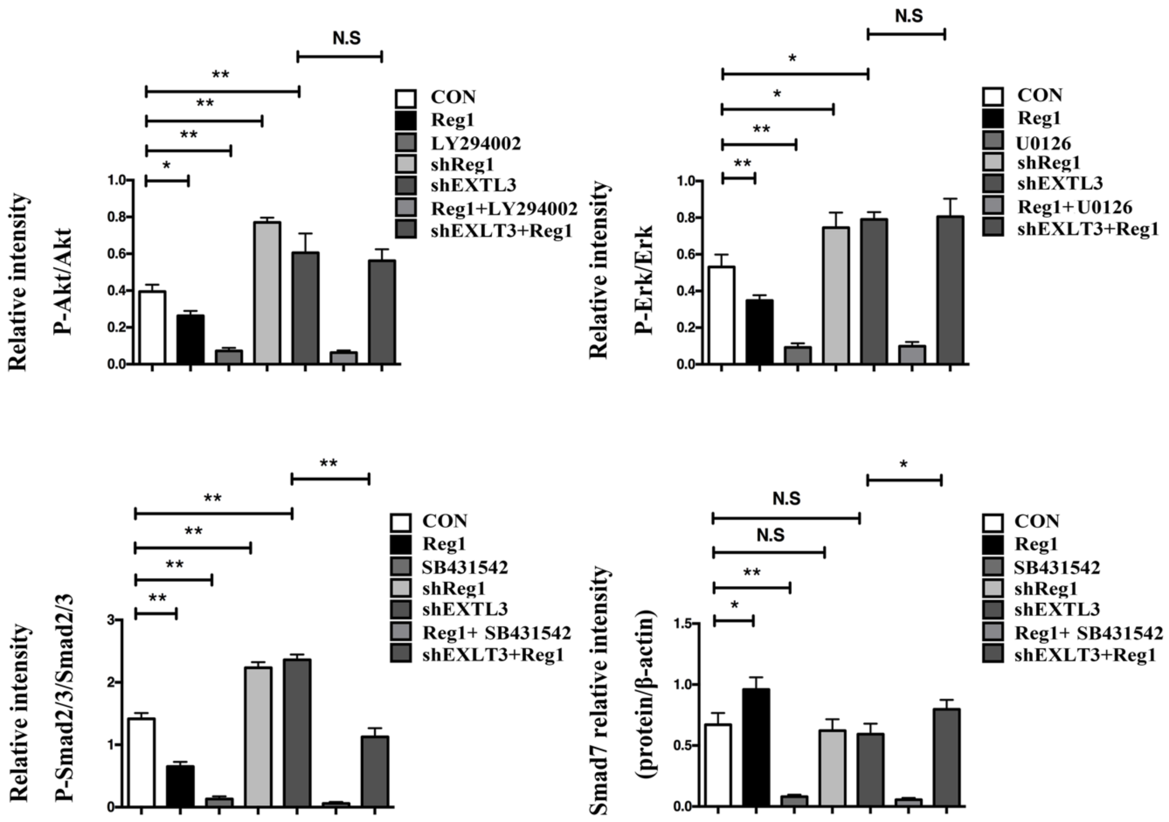

Supp Figure 7: Western blotting of db/db ISCs treated with rhReg1, U0126 (10uM), LY-294002 (10uM), SB431542 (10uM), sh-Reg1, sh-EXLT3 and sh-EXTL3+Reg1 by Erk1/2, P-Erk1/2, Akt, P-Akt, Smad2/3, P-Smad2/3 and Smad7 antibody. Data are expressed as mean ± SE (n = 3), * P < 0.05, ** P < 0.01.
